# Olive Pomace Phenolic Compounds and Extracts Can Inhibit Inflammatory- and Oxidative-Related Diseases of Human Ocular Surface Epithelium

**DOI:** 10.3390/antiox10071150

**Published:** 2021-07-20

**Authors:** Nikolaos Katsinas, Soraya Rodríguez-Rojo, Amalia Enríquez-de-Salamanca

**Affiliations:** 1 Institute of Applied Ophthalmobiology (IOBA), Campus Miguel Delibes, University of Valladolid (UVa), Paseo de Belén 17, 47011 Valladolid, Spain; nkatsinas@ioba.med.uva.es; 2 High Pressure Processes Group, Department of Chemical Engineering and Environmental Technology, School of Engineering, University of Valladolid (UVa), Dr. Mergelina str., 47011 Valladolid, Spain; soraya.rodriguez@uva.es; 3 Biomedical Research Networking Center in Bioengineering, Biomaterials and Nanomedicine (CIBER-BBN), Av. Monforte de Lemos, 3-5, 28029 Madrid, Spain

**Keywords:** agro-industrial by-product, sustainability, cornea and conjunctiva, ocular surface, olive pomace phenolic extracts, oleuropein, hydroxytyrosol, inflammation, dry eye, cellular antioxidant activity

## Abstract

Oxidative- and inflammatory-related ocular surface diseases have high prevalence and are an emerging issue in ophthalmology. Olive pomace (OP) is the olive oil’s industry main by-product, and is potentially environmentally hazardous. Nevertheless, it contains phenolic compounds with important bioactivities, like oleuropein (OL) and hydroxytyrosol (HT). The antioxidant and anti-inflammatory effects of four OP extracts (CONV, OPT(1–3)), pure OL and HT, and mixtures thereof were screened on human corneal (HCE) and conjunctival epithelial (IM-ConjEpi) cells. CONV was conventionally extracted, while OPT(1–3) were produced by pressurized liquid extraction. Thanks to their improved activity, CONV and OPT3 (HT-enriched) were selected for dose-dependent studies. Cells were stimulated with tumor necrosis factor-α or ultraviolet-B radiation, measuring interleukin (IL)-1β, IL-6, IL-8, and IL-17A as well as interferon γ-induced protein [IP]-10 secretion or intracellular ROS production, respectively. On HCE, both extracts and HT inhibited the secretion of most measured ILs, demonstrating a strong anti-inflammatory effect; while in IM-ConjEpi, all samples decreased IP-10 secretion. Moreover, HT, OL, and both extracts showed strong dose-dependent antioxidant activity in both cell lines. Compared with CONV, OPT3 was active at lower concentrations, demonstrating that intensified extraction techniques are selective towards targeted biomarkers. Hence, a high-value application as potential ocular surface therapy was proposed for the OP valorization.

## 1. Introduction

Ocular inflammation can occur in many different parts of the eye and represents an emerging issue in ophthalmology [1]. It is involved in several ocular diseases, such as age-related macular degeneration, macular edema, and retinal vein occlusion [1]. Oxidative stress is also involved in the pathophysiology of many ocular diseases [2], like age-related macular degeneration [3,4], cataract [5], conjunctivochalasis [6], or ocular allergy [7]. Dry eye syndrome (DES) is a multifunctional disorder, in which inflammation and oxidative stress of the lacrimal functional unit (LFU) are considered the major pathophysiologic mechanisms. LFU dysfunction can lead to an abnormal tear film composition, and thus to an unstable precorneal film layer [7,8,9]. It appears with a high prevalence, ranging from 5 to 50% depending on the population [10], and considerably affects the patients’ quality of life. DES also causes an important economic burden, not only owing to the direct treatment and medical costs, but also because of the indirect costs derived by productivity loss at work [11]. The new treatment approaches for DES include artificial tears, mucin secretagogues, anti-inflammatory drugs like cyclosporine and rebamipide, and natural or chemically synthetized antioxidants [12]. Nowadays, the potential benefits of natural phenolic compounds conferring to ocular health improvement are gaining increasing interest; Abengózar-Vela et al. [13,14] showed the beneficial effects of wine-derived flavonoids and stilbenes for the treatment of inflammatory ocular surface diseases.

In the search for new sources of natural phenolic compounds with demonstrated antioxidant and anti-inflammatory activities, the olive pomace (OP) has been highlighted [15]. Additionally, the OP is the main by-product produced during the olive oil and table olives’ production. It has a high organic load and phenolic content, it is produced in big quantities, and it is stored in open-air bags [16,17,18]. Therefore, it generates a remarkable environmental concern, while its valorization as source of phenolic bio-active compounds is of chief importance for the sustainable growth of related industries. Among the phenolic compounds present in OP, simple phenols, such as hydroxytyrosol (HT), and secoiridoids, such as oleuropein (OL), are the main chemical classes [19,20]. Several in vitro and in vivo studies support the antioxidant, anti-inflammatory, antiatherogenic, cardioprotective, antihypertensive, hypoglycemic, antimicrobial, antiviral, cytostatic, molluscicidal, and endocrinal activities of OL, the major olive bio-phenol [21,22,23]. HT is a simple phenol that can also be obtained after the hydrolysis of OL during oil extraction [24]. For HT, anti-microbial, chemoprotective, skin-bleaching, antioxidant, anti-inflammatory, antiatherogenic, and cardioprotective activities have been reported [21,22,24,25,26,27]. HT derivates, present not only in the OP, but also in the olive mill wastewaters, have also demonstrated remarkable antioxidant and anti-inflammatory activities [28,29]. In addition, some studies propose HT as a possible treatment for neovascular age-related macular degeneration, protecting also from retinal oxidative stress-induced mitochondrial dysfunction and apoptosis [30,31,32,33,34]. Besides, González-Correa et al. [35] proved the neuroprotective effect of HT in diabetic retinopathy, while OL seems to prevent intraocular pressure elevation in vivo [36]. Recently, Mauro et al. [37] showed the anti-inflammatory and antioxidant activities of a polyphenolic fraction from olive mill wastewaters on a Statens Seruminstitut Rabbit Cornea cell line. However, to the best of our knowledge, an extended pharmacological study on the effect of the pure OL and HT or crude olive extracts on human ocular surface epithelial cells has not yet been performed.

The aim of this work was to evaluate the potential use of extracts from OP as a possible treatment for oxidative and inflammatory-related ocular surface diseases. Hence, crude extracts derived from OP, together with OL, HT, and their combination (OL + HT), were tested in vitro on human ocular surface epithelial cells to determine their antioxidant and anti-inflammatory activity. Two different ocular surface cell lines were selected, using as stimuli tumor necrosis factor (TNF)-α to induce inflammation and ultraviolet (UV)-B light to induce oxidative stress. Cytokine/chemokine secretion (interleukin (IL)-1β, IL-6, IL-8, IL-17A, and interferon γ-induced protein (IP)-10) and intracellular reactive oxygen species (ROS) production were measured as surrogate markers of inflammation and oxidative stress, respectively. Preliminarily, cytotoxicity of extracts and compounds was also evaluated.

## 2. Materials and Methods

### 2.1. Plant Material

OP of Arbequina variety, from 2018 crop, was kindly offered by Oliduero (Medina del Campo, Spain). Characterization and storage conditions of the material are explained in detail by Katsinas et al. [38].

### 2.2. Materials

For the preparation of the phenolic extracts, MilliQ water was obtained from a Millipore unit, ethanol (EtOH) non denaturalized (99.9%) from Dávila Villalobos S.L. (Valladolid, Spain), CO_2_ (99.95%) from Carburos Metálicos (Barcelona, Spain), and N_2_ (99.996%) from Linde Gas (Dublin, Ireland). HT (≥98%) and OL (≥98%) were supplied by Extrasynthese (Genay, France). For the cell-based assays, plastic culture flasks, plates, tips and pipettes, Dulbecco’s modified Eagle’s medium/Nutrient Mixture F-12 (DMEM/F-12) + GlutaMax, DMEM/F12 without phenol red, Dulbecco’s phosphate buffered saline (DPBS), fetal bovine serum (FBS), human epithelial growth factor (EGF), human insulin, penicillin, streptomycin, and bicinchoninic acid (BCA) assay kit were purchased from Thermo Fisher Scientific (Rockford, IL, USA); cytokine TNF-α from bioNova scientific (Fremont, CA, US); bovine insulin, 20,70-dichlorodihydrofluorescein diacetate (H2DCF-DA), 2,3-Bis-(2-Methoxy-4-Nitro-5-Sulfophenyl)-2H-Tetrazolium-5-Carboxanilide (XTT) and 5-methylphenazinium methyl sulfate (PMS) reagents, dimethyl sulfoxide (DMSO), and Milliplex Human Cytokine/Chemokine HCYTOMAG-60K-5 plex Magnetic Kit (IL-1beta, IL-6, IL-8/CXCL8, IL-17A, and IP-10) from Merck Life Science (St. Louis, MO, USA).

### 2.3. Preparation and Characterization of Phenolic Extracts

The phenolic extraction procedure and the characterization of the extracts were performed as previously described [38].

Briefly, all extracts were characterized in terms of extraction yield (EY) (expressed as mg of dry extract (DE)/g of DRY OP); total phenolic content (TPC) by the Folin–Ciocalteu method (expressed as mg of gallic acid equivalents (GAE)/g of DE); chemical antioxidant capacity by the oxygen radical absorbance capacity (ORAC) method (expressed as mmol of Trolox equivalents (TE)/g of DE); and extract richness in HT, OL, and Tyrosol (TY) (expressed as mg of compound/g of DE) by HPLC-DAD (Waters e2695 separation module with autosampler and quaternary pump, connected to a Waters 2998 photodiode array detector). The injection volume used was 20 μL and the chromatograms were obtained at 280 nm. The column used was a C18 Mediterranean Sea column (250 × 4.6 mm, 5 μm) (Teknokroma Analítica S.A., Barcelona, Spain) at 35 °C connected with an OptiGuard 1 mm guard column (Sigma-Aldrich, San Luis, Misuri, USA). A gradient system of two eluents (methanol and acidified water to pH = 3 with phosphoric acid) was applied, with a flow of 1 mL/min [38]. Empower^®^ 3 software (Waters^®^, Ireland, UK) was used for data acquisition and processing.

The conventional extract (CONV) was produced using freeze-dried OP (FD-OP) and following the conventional solid–liquid phenolic extraction procedure of phenolic compounds (conditions: temperature = 70 °C, % of EtOH in water = 50.0%, solid/liquid ratio = 0.5 g_RAW OP_/mL_SOLVENT_, extraction time = 1 h, at atmospheric pressure). To produce the optimized extracts (OPT), the FD-OP was defatted with supercritical carbon CO_2_ (scCO_2_). Following this, hydroalcoholic extracts were produced by pressurized liquid extraction (PLE) at three different conditions, previously optimized: OPT1 (at temperature = 66.0 °C, % of EtOH in water = 10.0%, and solid/liquid ratio = 0.8 g_RAW OP_/mL_SOLVENT_), which had the highest ORAC antioxidant activity (ORAC-AA); OPT2 (at temperature = 66.0 °C, % of EtOH in water = 92.0%, and solid/liquid ratio = 0.8 g_RAW OP_/mL_SOLVENT_), which had the highest concentration in OL; and OPT3 (at temperature = 184.0 °C, % of EtOH in water = 90.0%, and dolid/liquid ratio = 0.8 g_RAW OP_/mL_SOLVENT_), which was enriched in HT, TY, and TPC. For all PLE conditions, an extraction time of 20 min was applied.

### 2.4. Cell-Based Assays

#### 2.4.1. Cell Culture

Two ocular surface epithelial cell lines were selected to perform the experiments: human corneal epithelial (HCE) cells and the immortalized human conjunctival epithelial (IM-ConjEpi) cells.

The HCE cell line was kindly given by Dr. Arto Urti (University of Helsinki, Finland). It is an SV40- Large T Antigen immortalized human corneal epithelial cell line, already characterized [39]. HCE cells were cultured in DMEM/F-12 + GlutaMax supplemented with 10% FBS, 10 ng/mL EGF, 5 μg/mL human insulin, and antibiotics (100 U/mL of penicillin and 0.1 mg/mL of streptomycin). The passages used were from 30 to 40.

The IM-ConjEpi cell line is an SV40- Large T Antigen immortalized human conjunctival epithelial cell line, purchased from Innoprot (Derio, Spain. Ref. P10870-IM). IM-ConjEpi cells were cultured in DMEM/F-12 + GlutaMax supplemented with 10% FBS, 10 ng/mL EGF, 1 μg/mL bovine insulin, and antibiotics (5000 U/mL of penicillin and 5000 μg/mL of streptomycin). The passages used were from 5 to 15.

The cells were cultured in an incubator with a temperature of 37 °C and a ratio of 5:95—CO_2_/air atmosphere. Their medium was changed every second day and daily observations were performed using a phase-contrast microscope.

#### 2.4.2. Preparation of Phenolic Solutions

HT and OL were dissolved in culture medium (DMEM/F-12 + GlutaMax, without any supplement), and CONV, OPT1, OPT2, and OPT3 in 0.4% EtOH in culture medium, on the same day of the experiment. Then, serial dilutions were performed, reaching the final desired concentrations: 1 to 150 μM for HT, 5 to 325 μM for OL, 0.05 to 0.80 mg/mL for CONV, 0.05 and 0.40 mg/mL for OPT1, 0.05 and 0.40 mg/mL for OPT2, and 0.005 to 0.600 mg/mL for OPT3. A mixture of 5 μM of OL with 10, 25, or 50 μM of HT and a mixture of 10 μM of OL with 50 μM of HT were prepared by mixing the double concentration of each compound in equal volumes.

#### 2.4.3. Cell Viability Assay

The XTT assay was selected to test the in vitro toxicity of HT, OL, and four OP extracts (CONV, OPT1, OPT2, and OPT3). HCE and IM-ConjEpi cells were placed in 96-well plates (31,250 HCE cells/cm^2^ and 43,750 IM-ConjEpi cells/cm^2^) and, when the confluence reached 90% of the well surface, their culture medium was replaced by DMEM/F-12 + GlutaMax without any supplement. After 24 h, the medium was discarded and the cells were treated with the different concentrations of OL (200, 250, and 300 μM for both cell lines, and 325 μM for HCE), HT (50, 100, and 150 μM), OL + HT (5 μM OL + 50 μM HT, 10 μM OL + 50 μM HT), CONV (0.20, 0.40, 0.60, and 0.80 mg/mL), OPT1 (0.20, 0.40, 0.60, and 0.80 mg/mL), OPT2 (0.20, 0.40, 0.60, and 0.80 mg/mL), and OPT3 (0.200 and 0.400 mg/mL for both cell lines, and 0.600 mg/mL for HCE). Vehicle-treated cells were used as control and 0.001% of benzalkonium chloride was used as positive control (data not shown). The cells were incubated in the presence of the treatments for 24 h at 37 °C. Subsequently, supernatants were removed and DMEM/F-12 without phenol red was placed. Then, 10 μL of 3 mg PMS/mL DPBS was mixed with 4 mL of 1 mg XTT/mL DMEM/F-12 without phenol red and the prepared solution was directly added to the cells. The cells were incubated at 37 °C for 4 h. After the incubation, the absorbance was measured at 450 nm and 660 nm by a UV/vis spectrophotometer (SpectraMax M5; Molecular Devices, Sunnyvale, CA, USA). Three independent experiments and six replicates for each treatment per experiment were performed.

#### 2.4.4. Anti-Inflammatory Activity

##### Cell Cytokine Stimulation

The protocol of cell cytokine stimulation was performed as previously described [13]. Briefly, HCE and IM-ConjEpi cells were seeded in 24-well plates (350,000 HCE cells/cm^2^ and 550,000 IM-ConjEpi cells/cm^2^) and, when the confluence reached 90% of the well surface, their culture medium was replaced by DMEM/F-12 + GlutaMax without any supplement. After 24 h, the medium was discarded and the cells were treated with the different concentrations of OL (5, 10, 25, 50, 100, 150, 200, 250, and 300 μM), HT (1, 5, 10, 25, 50, and 100 μM for both cell lines, and 150 μM for HCE), OL + HT (5 μM OL + 10 μM HT, 5 μM OL + 25 μM HT, and 5 μM OL + 50 μM HT), CONV (0.05, 0.10, 0.20, 0.40, 0.60, and 0.80 mg/mL), OPT1 (0.40 mg/mL, tested only in HCE), OPT2 (0.40 mg/mL, tested only in HCE), and OPT3 (0.005, 0.025, 0.050, 0.100, 0.200, and 0.400 mg/mL) for 2 h at 37 °C. Subsequently, the supernatants were discarded, and the cells were stimulated with 25 ng/mL TNF-α in the presence of the treatments for 24 h at 37 °C. Vehicle-treated cells and cells not stimulated with TNF-α were used as controls. After the incubation, the supernatants were collected, centrifuged at 18,800× *g* for 10 min and stored at −80 °C until use. Plates with adherent cells were also stored at −80 °C until protein extraction. Three independent experiments and two replicates for each treatment per experiment were performed.

##### Cell Cytokine Secretion Measurement

IL-1β, IL-6, IL-8, IL-17A, and IP-10 cytokines were measured in cell supernatants by a multiplex bead-based array, using a commercial Milliplex 5-plex Human Cytokine/Chemokine immunobead-based kit (HCYTOMAG-60K), as already described [40]. According to the manufacturer’s instructions, 25 μL of each supernatant was incubated at 4 °C overnight, in the presence of antibody-immobilized beads. After the incubation, the beads were washed, and a detection antibodies solution (biotinylated cytokine/chemokine antibodies) was added. The plate was incubated under shaking for 1 h at room temperature. Subsequently, a streptavidin-phycoerythrin solution was added and the plate was incubated at room temperature for 30 min, under shaking. Finally, the beads were washed and resuspended in sheath fluid. The plate was read in a Luminex 100-IS (Luminex Corporation, Austin, TX, USA). Calibration curves of each human cytokine/chemokine standard were performed, converting fluorescent to cytokine/chemokine concentration units (pg/mL) using the BeadView Software (Upstate, UK). The range of the standard curve for the cytokines/chemokines selected was 3.2 to 10,000 pg/mL. If a cytokine was detected outside the calibration curve area, it was replaced by the minimum (for low/not-detected values) or maximum (for high/extrapolated values) detectable value. Cytokine concentration data were normalized to corresponding protein content of each well, determined by the BCA protein assay [41], following the manufacturer’s instructions.

#### 2.4.5. Antioxidant Activity: UV-B Induced ROS Production Measurement

Intracellular ROS production after UV-B stimulation was determined by the H2DCF-DA dye assay, as previously described [13]. Briefly, HCE and IM-ConjEpi cells were seeded in 24-well plates (350,000 HCE cells/cm^2^ and 550,000 IM-ConjEpi cells/cm^2^) in complete culture medium. When the confluence reached 90% of the well surface, culture medium was replaced by DMEM/F-12 + GlutaMax without any supplement. After 24 h, the medium was discarded and the cells were treated with the different concentrations of OL (5, 10, 25, 50, 100, 150, 200, 250, and 300 μM), HT (1, 5, 10, 25, 50, and 100 μM for both cell lines, and 150 μM for HCE), OL + HT (5 μM OL + 10 μM HT, 5 μM OL + 25 μM HT, and 5 μM OL + 50 μM HT), CONV (0.05, 0.10, 0.20, 0.40, 0.60, and 0.80 mg/mL), OPT1 (0.05 mg/mL, tested only in HCE), OPT2 (0.05 mg/mL, tested only in HCE), and OPT3 (0.005, 0.025, 0.050, 0.100, 0.200, and 0.400 mg/mL) for 1 h at 37 °C. Subsequently, the supernatants were aspirated, and the cells were incubated with 10 μM H2DCF-DA solution for 30 min. Following this, the H2DCF-DA solution was discarded, and the cells were treated with the aforementioned treatments (at the same concentrations) and exposed to UV-B lamp (Bio-Rad, Inc., Hercules, CA, USA) for 15 s. Vehicle-treated cells and cells not stimulated with UV-B were used as controls. The cells were incubated in the presence of the treatments for 1 h at 37 °C, and then their intracellular fluorescence intensity was measured at 488 nmex and 522 nmem by a UV/vis spectrophotometer (SpectraMax M5; Molecular Devices, Sunnyvale, CA, USA). Data were normalized to the corresponding protein content in each well determined by the BCA protein assay [41], following the manufacturer’s instructions. Three independent experiments and two replicates for each treatment per experiment were performed.

### 2.5. Statistical Analysis

The data are presented as mean ± standard error of the mean (SEM). To study the homogeneity of variances, the Levene’s test was performed. One-way analysis of variances (ANOVA) with Tukey’s or Games–Howell post hoc test was applied to compare stimulated with non-stimulated cells, as well as for intergroup comparisons. *p*-values lower than 0.05 were considered statistically significant. The identification of the presence or absence of outliers was performed using ROUT analysis of the individual values. No outliers were removed from the analysis because of biological diversity or technical errors. To perform the statistical analysis, the SPSS software (SPSS 15.0; SPSS, Inc., Chicago, IL, USA) was used.

## 3. Results

### 3.1. Characterization of Phenolic Extracts

As already described [38], all selected OP extracts were characterized in terms of EY, TPC, and ORAC-AA, as well as extract richness in OL, HT, and TY. The results are presented in Table 1.

### 3.2. Effect of Phenolic Solutions on Cell Viability

**Figure 1:**
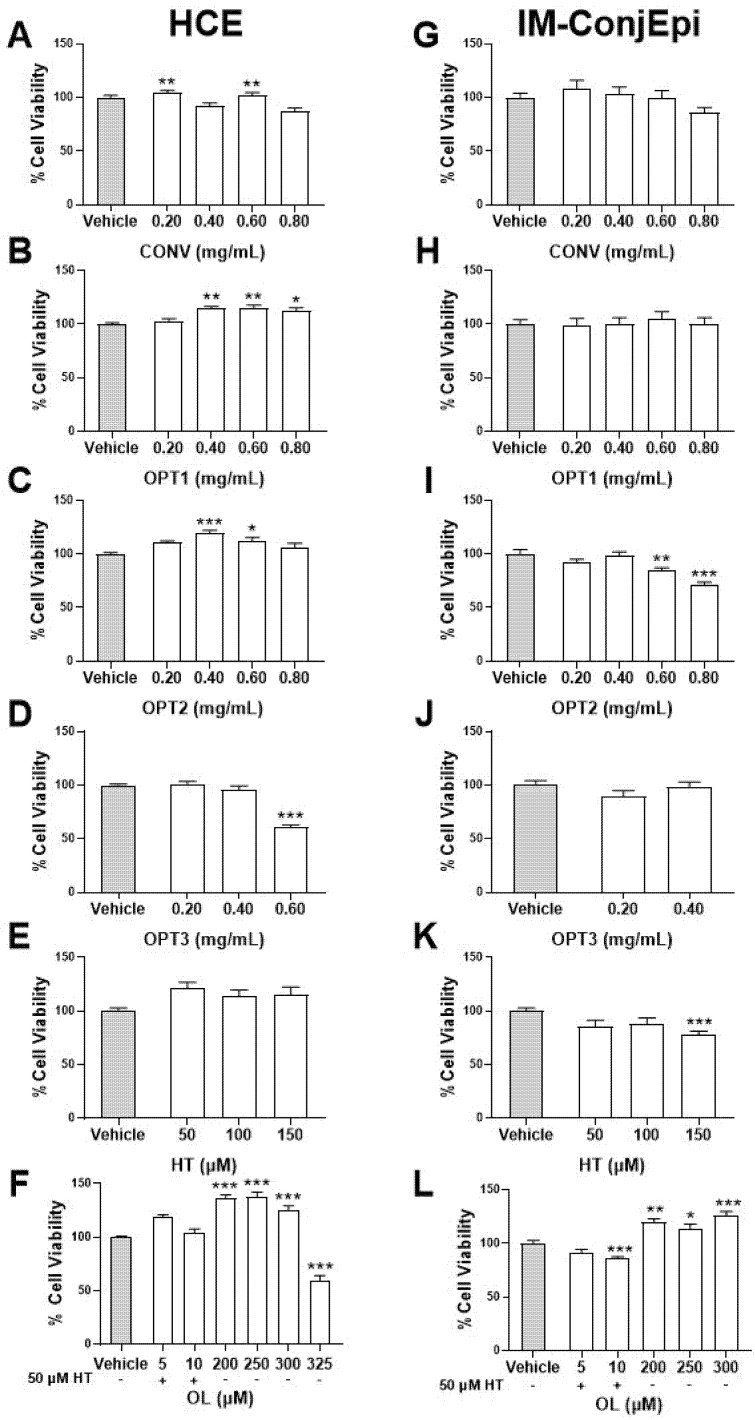
Cell viability assays of Hydroxytyrosol (HT), Oleuropein (OL), and their combination, together with the conventional (CONV) and the three optimized (OPT1, OPT2, and OPT3) olive pomace (OP) extracts on HCE (**A**–**F**) and IM-ConjEpi (**G**–**L**) cells. Cells were treated for 24 h with HT (50, 100, and 150 μM), OL (200, 250, and 300 μM for both cells lines, and 325 μM for HCE), OL + HT (5 μM of OL + 50 μM of HT and 10 μM of OL + 50 μM of HT), CONV (0.20, 0.40, 0.60, and 0.80 mg/mL), OPT1 (0.20, 0.40, 0.60, and 0.80 mg/mL), OPT2 (0.20, 0.40, 0.60, and 0.80 mg/mL), OPT3 (0.20 and 0.40 mg/mL for both cell lines, and 0.60 mg/mL for HCE), or vehicle (cell culture medium for HT and OL or 0.4% ethanol for the extracts). The in vitro toxicity was performed using the XTT assay. For CONV and OPT1, none of the concentrations tested were considered toxic, neither for HCE (**A**,**B**, respectively) nor for IM-ConjEpi (**G**,**H**, respectively). OPT2 is nontoxic up to 0.80 mg/mL for HCE (**C**) and 0.40 mg/mL for IM-ConjEpi (**I**), whereas up to 0.40 mg/mL of OPT3 is nontoxic for both cell lines (**D**,**J**). For HT, only 150 μM decreased cell viability on IM-ConjEpi (**K**), while all concentrations were safe for HCE (**E**). For OL, only 325 μM was considered toxic for HCE (**F**), while all concentrations tested on IM-ConjEpi were considered nontoxic (**L**). For the OL + HT mixture, 10 μM OL + 50 μM HT was toxic for IM-ConjEpi (**L**), while both mixtures were safe for HCE (**F**). Data are presented as percentage of cell viability of three independent experiments (performed in sextuplicate) ± SEM. * *p* < 0.05, ** *p* < 0.01, *** *p* < 0.001, compared with vehicle-treated cells. Vehicle: Cell culture medium (**A**–**D** and **G**–**J**) and 0.4% EtOH (**E**,**F**,**K**,**L**). Benzalkonium chloride was used as positive control (data not shown).

HCE and IM-ConjEpi cells were exposed for 24 h to different concentrations of HT, OL, and the four selected OP extracts (CONV, OPT1, OPT2, and OPT3) to evaluate their in vitro effect on cell viability. Figure 1 presents their cytotoxicity as a percentage of viable HCE (A–F) and IM-ConjEpi (G–L) cells. No toxic effect was observed for 0.4% EtOH vehicle-treated cells compared with control (culture medium-treated cells, data not shown). Regarding the OP extracts, CONV and OPT1 did not decrease the cell viability significantly in any of the selected ocular surface cell lines. OPT2 in IM-ConjEpi cells and OPT3 in HCE cells showed a significant cytotoxic effect from 0.60 mg/mL. Regarding the individual phenolic compounds, only 150 μM of HT and 325 μM of OL significantly decreased the viability of IM-ConjEpi and HCE cells, respectively. Moreover, the combination OL + HT showed no cytotoxic effect on cells up to 5 μM of OL + 50 μM of HT (Figure 1F,L).

### 3.3. Anti-Inflammatory Activity of Phenolic Solutions

The anti-inflammatory activity of the non-toxic concentrations of the phenolic solutions was tested on the TNF-α-induced cytokine/chemokine secretion by HCE and IM-ConjEpi cells. IL-6, IL-8, IL-1β, and IP-10 secretion was significantly stimulated by 25 ng/mL TNF-α on both cell lines. In the case of IL-1β, the stimulation was not sufficient in two experiments, while IL-17A production was not increased by the stimulus selected neither on HCE, nor on IM-ConjEpi cells (data not shown).

**Figure 2:**
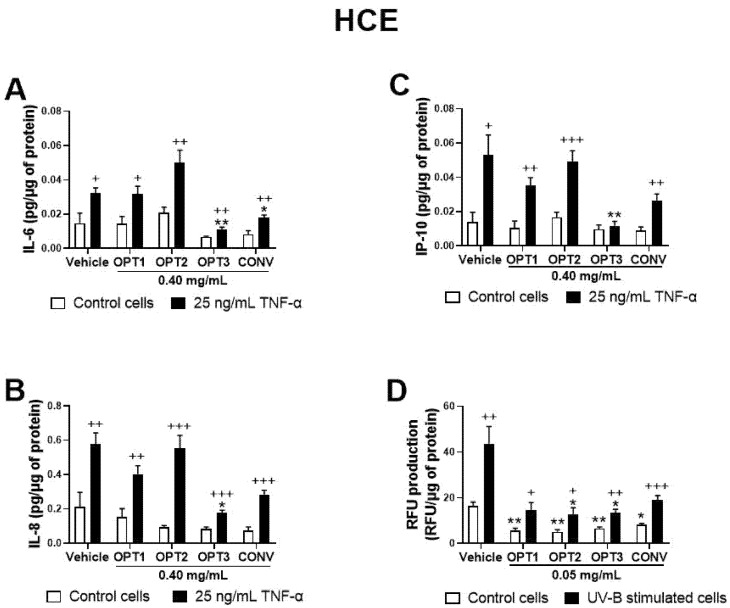
Screening of the anti-inflammatory and antioxidant activity of the conventional (CONV) and the three optimized (OPT1, OPT2, and OPT3) Olive Pomace (OP) extracts on TNF-α-induced cytokine release and UV-B-induced reactive oxygen species (ROS) production by HCE cells, respectively. For cell cytokine stimulation, cells were pre-treated with vehicle (0.4% ethanol) or 0.40 mg/mL of CONV, OPT1, OPT2, and OPT3 for 2 h. Following this, they were stimulated with 25 ng/mL TNF-α in the presence of the treatments for 24 h (**A**–**C**, black bars). Vehicle-treated-TNF-α stimulated cells and cells not stimulated with TNF-α (**A**–**C**, white bars) were used as control. IL-6, IL-8, and IP-10 were measured in cell supernatants by a multiplex bead-based array. OPT3 inhibited all measured cytokines/chemokines (**A**–**C**), while CONV significantly decreased IL-6 (**A**). For the UV-B-induced ROS production, cells were pre-treated with vehicle (0.4% ethanol) or 0.05 mg/mL of CONV, OPT1, OPT2 and OPT3 for 1 h. Subsequently, cells were incubated with 10 μM H2DCF-DA solution for 30 min, and then treated with the treatments and exposed to 107.25 mJ/cm2 UV-B light (**D**, black bars). After 1 h of incubation, intracellular fluorescence intensity was measured. Vehicle-treated-UV-B stimulated cells and cells not stimulated with UV-B (**D**, white bars) were used as control. OPT2 and OPT3 decreased ROS levels significantly. Data are presented as picograms (pg) of cytokine/chemokine (for cell cytokine stimulation) or relative fluorescence units (RFUs) (for ROS production) normalized to micrograms (μg) of total protein for three independent experiments (performed in duplicate) ± SEM. * *p* < 0.05, ** *p* < 0.01, compared with vehicle-treated cells; + *p* < 0.05, ++ *p* < 0.01, +++ *p* < 0.001, compared with control cells. Vehicle: 0.4% EtOH.

**Figure 3:**
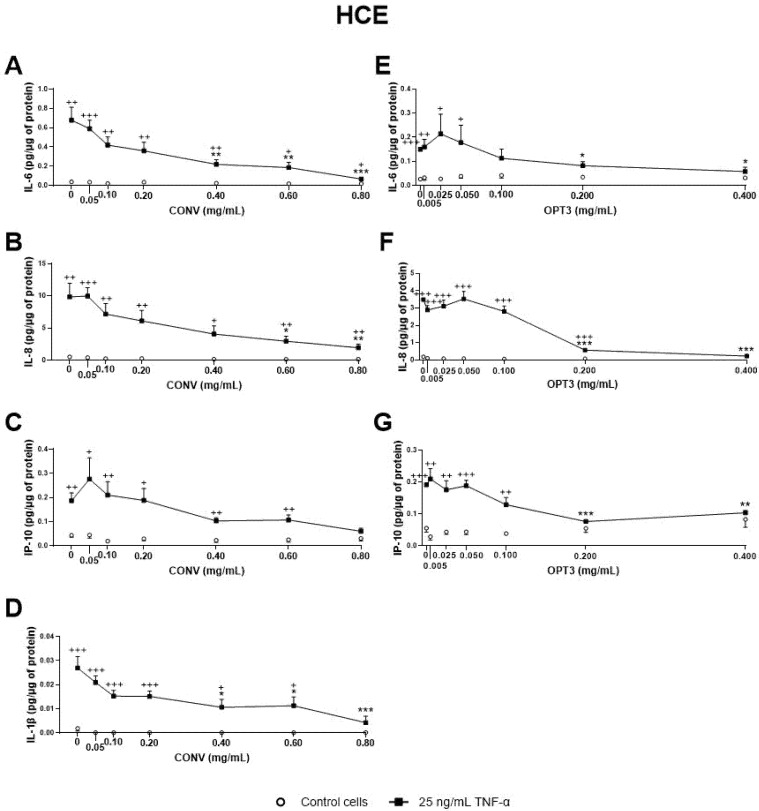
Effect of the conventional (CONV) and the selected optimized (OPT3) olive pomace (OP) extracts on TNF-α-induced cytokine release by HCE cells. Cells were pre-treated with CONV (0.05, 0.10, 0.20, 0.40, 0.60, and 0.80 mg/mL), OPT3 (0.005, 0.025, 0.050, 0.100, 0.200, and 0.400 mg/mL), or vehicle (0.4% ethanol) for 2 h. Following this, they were stimulated with 25 ng/mL TNF-α in the presence of the treatments for 24 h (black squares). Vehicle-treated-TNF-α stimulated cells and cells not stimulated with TNF-α (white circles) were used as control. IL-6, IL-8, IP-10, and IL-1β were measured in cell supernatants by a multiplex bead-based array. TNF-α failed to stimulate IL-1β in the experiment performed for OPT3. CONV significantly decreased IL-6 levels from 0.40 mg/mL (**A**), IL-8 levels from 0.60 mg/mL (**B**), and IL-1β levels from 0.40 mg/mL (**D**). For TNF- α stimulated cells, IP-10 production was not decreased significantly by CONV. Nevertheless, no significant differences were observed between stimulated and non-stimulated cells at 0.80 mg/mL (**C**). OPT3 inhibited IL-6 (**E**), IL-8 (**F**), and IP-10 (**G**) secretion from 0.200 mg/mL significantly. Data are presented as picograms (pg) of cytokine/chemokine per micrograms (μg) of total protein for three independent experiments (performed in duplicate) ± SEM. * *p* < 0.05, ** *p* < 0.01, *** *p* < 0.001, compared with vehicle-treated-TNF-α stimulated cells; + *p* < 0.05, ++ *p* < 0.01, +++ *p* < 0.001, compared with control cells.

**Figure 4:**
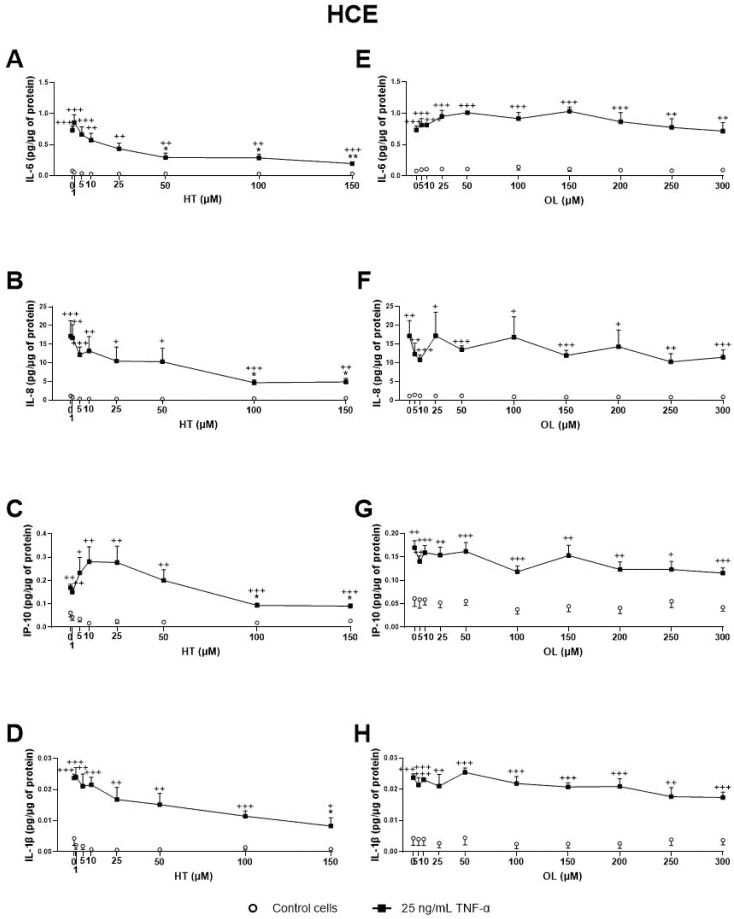
Effect of Hydroxytyrosol (HT) and Oleuropein (OL) on TNF-α-induced cytokine release by HCE cells. Cells were pre-treated with HT (1, 5, 10, 25, 50, and 100 μM for both cell lines, and 150 μM for HCE), OL (5, 10, 25, 50, 100, 150, 200, 250, and 300 μM) or vehicle (cell culture medium) for 2 h. Following this, they were stimulated with 25 ng/mL TNF-α in the presence of the treatments for 24 h (black squares). Vehicle-treated-TNF-a stimulated cells and cells not stimulated with TNF-α (white circles) were used as control. IL-6, IL-8, IP-10, and IL-1β were measured in cell supernatants by a multiplex bead-based array. HT significantly decreased IL-6 (**A**), IL-8 (**Β**), IP-10 (**C**), and IL-1β (**D**) secretion at 50, 100, 100, and 150 μM, respectively. OL failed to inhibit any of the cytokines/chemokines measured (**E**–**H**). Data are presented as picograms (pg) of cytokine/chemokine per micrograms (μg) of total protein for three independent experiments (performed in duplicate) ± SEM. * *p* < 0.05, ** *p* < 0.01, compared with vehicle-treated-TNF-α stimulated cells; + *p* < 0.05, ++ *p* < 0.01, +++ *p* < 0.001, compared with control cells.

**Figure 5:**
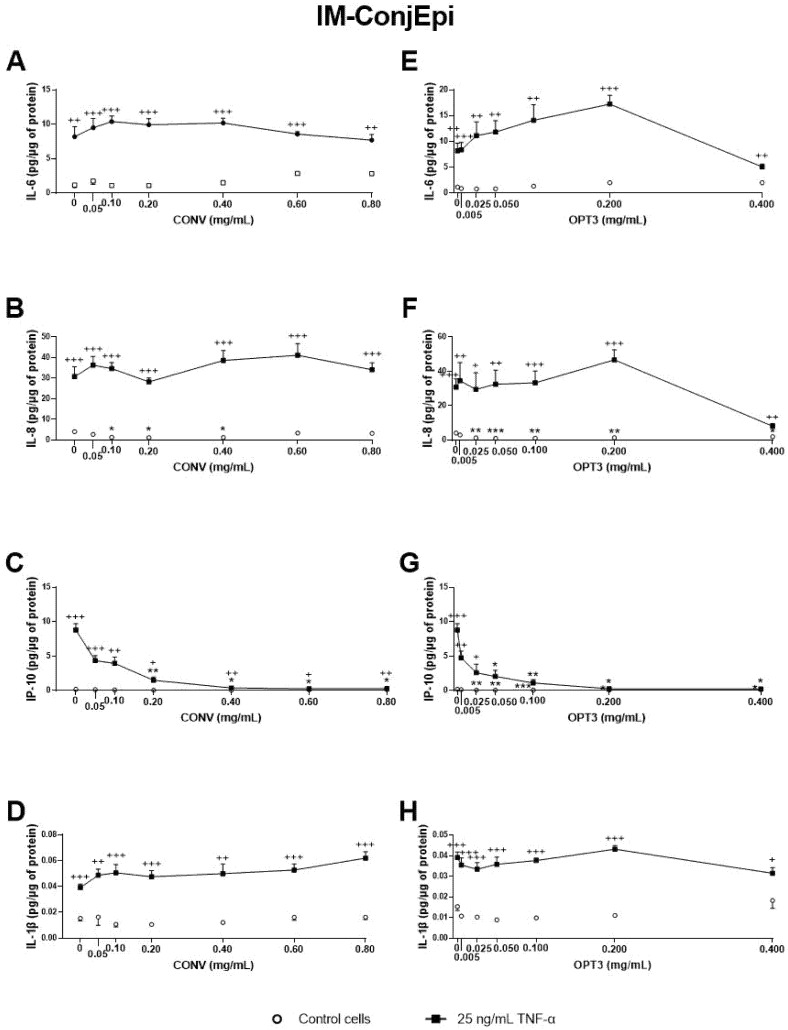
Effect of the conventional (CONV) and the selected optimized (OPT3) olive pomace (OP) extracts on TNF-α-induced cytokine release by IM-ConjEpi cells. Cells were pre-treated with CONV (0.05, 0.10, 0.20, 0.40, 0.60, and 0.80 mg/mL), OPT3 (0.005, 0.025, 0.050, 0.100, 0.200, and 0.400 mg/mL) or vehicle (0.4% ethanol) for 2 h. Following this, they were stimulated with 25 ng/mL TNF-α in the presence of the treatments for 24 h (black squares). Vehicle-treated-TNF-α stimulated cells and cells not stimulated with TNF-α (white circles) were used as control. IL-6, IL-8, IP-10, and IL-1β were measured in cell supernatants by a multiplex bead-based array. CONV and OPT3 reduced IP-10 levels at 0.20 mg/mL (**C**) and 0.05 mg/mL (**G**), respectively. IL-8 levels at baseline cells were decreased by CONV and OPT3 at 0.10 mg/mL (**B**) and 0.025 mg/mL (**F**), respectively. No significant inhibition was observed for IL-6 and IL-1β, neither by CONV (**A**,**D**, respectively) nor by OPT3 (**E**,**H**, respectively). Data are presented as picograms (pg) of cytokine/chemokine per micrograms (μg) of total protein for three independent experiments (performed in duplicate) ± SEM. * *p* < 0.05, ** *p* < 0.01, *** *p* < 0.001, compared with vehicle-treated-TNF-α stimulated cells; + *p* < 0.05, ++ *p* < 0.01, +++ *p* < 0.001, compared with control cells.

**Figure 6:**
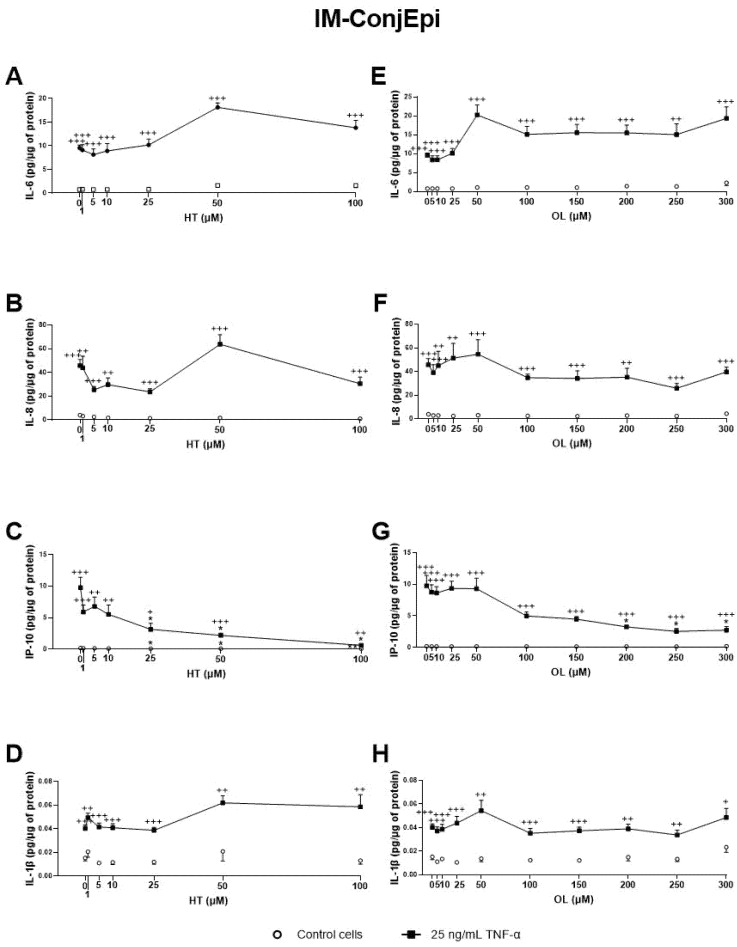
Effect of Hydroxytyrosol (HT) and Oleuropein (OL) on TNF-α-induced cytokine release by IM-ConjEpi cells. Cells were pre-treated with HT (1, 5, 10, 25, 50, and 100 μM), OL (5, 10, 25, 50, 100, 150, 200, 250, and 300 μM), or vehicle (cell culture medium) for 2 h. Following this, they were stimulated with 25 ng/mL TNF-α in the presence of the treatments for 24 h (black squares). Vehicle-treated-TNF-α stimulated cells and cells not stimulated with TNF-α (white circles) were used as control. IL-6, IL-8, IP-10, and IL-1β were measured in cell supernatants by a multiplex bead-based array. HT and OL reduced IP-10 levels at 25 μM (**C**) and 200 μM (**G**), respectively. No significant inhibition was observed for IL-6, IL-8, and IL-1β, neither by HT (**A**,**B**,**D**, respectively) nor by OL (**E**,**F**,**H**, respectively). Data are presented as picograms (pg) of cytokine/chemokine per micrograms (μg) of total protein for three independent experiments (performed in duplicate) ± SEM. * *p* < 0.05, compared with vehicle-treated-TNF-α stimulated cells; + *p* < 0.05, ++ *p* < 0.01, +++ *p* < 0.001, compared with control cells.

As a first preliminary screening, four OP extracts, CONV, OPT1, OPT2, and OPT3, were tested on HCE at the same concentration (0.40 mg/mL) to compare their activity. Figure 2A–C shows their effect on IL-6, IL-8, and IP-10 secretion for this cell line. IL-1β was not detected in this experiment (data not shown). CONV was able to significantly decrease IL-6 secretion by HCE cells, while optimized extract OPT3 significantly inhibited all the measured cytokines/chemokines. Moreover, the effect of OPT3 on IP-10 secretion was so strong that, in its presence, no significant differences were observed between TNF-α-stimulated and non-stimulated cells. Comparing the results among the four extracts, OPT3 demonstrated significantly higher anti-inflammatory activity compared with the rest of the extracts for IL-6 (*p*-value = 0.032 with OPT1; *p*-value = 0.014 with OPT2; *p*-value = 0.045 with CONV) and IL-8 (*p*-value = 0.026 with OPT1; *p*-value = 0.022 with OPT2; *p*-value = 0.032 with CONV). Furthermore, compared with OPT2, OPT3 had stronger inhibitory activity for IP-10 (*p*-value = 0.004), and CONV for IL-6 (*p*-value = 0.031). Therefore, according to the preliminary assay results, CONV and OPT3 were the only two extracts selected for dose-dependent studies, along with HT and OL, in both cell lines (shown in Figure 3, Figure 4, Figure 5 and Figure 6).

Regarding the OP extracts’ effect on HCE cells, as shown in Figure 3, CONV significantly decreased IL-6, IL-8, and IL-1β TNF-α stimulated secretion by cells from 0.40, 0.60, and 0.40 mg/mL, respectively (Figure 3A–D). It is also important to note that, at a concentration of 0.8 mg/mL, no statistically significant differences in IL-1β and IP-10 levels were observed between TNFα-stimulated and non-stimulated cells, demonstrating a preventive anti-inflammatory effect. On the other hand, OPT3 significantly decreased stimulated IL-6, IL-8, and IP-10 secretion by HCE cells from 0.200 mg/mL, whereas IL-1β was not detected in this experiment (Figure 3E–G). Further, OPT3 prevented IL-6, IL-8, and IP-10 production at 0.100, 0.400, and 0.200 mg/mL, respectively. Regarding pure phenolic compounds, HT significantly inhibited the secretion of IL-6, IL-8, and IP-10 from 50, 100, and 100 μM (Figure 4A–C), respectively, while only 150 μM of HT was able to decrease IL-1β levels (Figure 4D). However, OL did not demonstrate any significant anti-inflammatory effect on HCE cells (Figure 4E–H).

Additionally, the effect of CONV and OPT3 extracts and of HT and OL on cytokine secretion by IM-ConjEpi cells is shown in Figure 5; Figure 6, respectively. CONV, OPT3, HT, and OL significantly decreased IP-10 levels starting from 0.20 mg/mL (Figure 5C), 0.05 mg/mL (Figure 5G), 25 μM (Figure 6C), and 200 μM (Figure 6G), respectively. In the case of OPT3, from 0.050 mg/mL, no significant differences were observed for IP-10 levels between TNF-α- stimulated and non-stimulated cells, while from 0.025 mg/mL, IP-10 levels also decreased in non-stimulated cells (Figure 5G). The preventing anti-inflammatory effect of OPT3 was also observed for IL-8, whose levels in the baseline cells decreased significantly from 0.025 mg/mL (Figure 5F). Cytokine secretion inhibition effect in non-stimulated cells was also observed by 0.10–0.40 mg/mL of CONV for IL-8 (Figure 5B), and by HT from 25 μM for IP-10 (Figure 5C).

**Figure 7:**
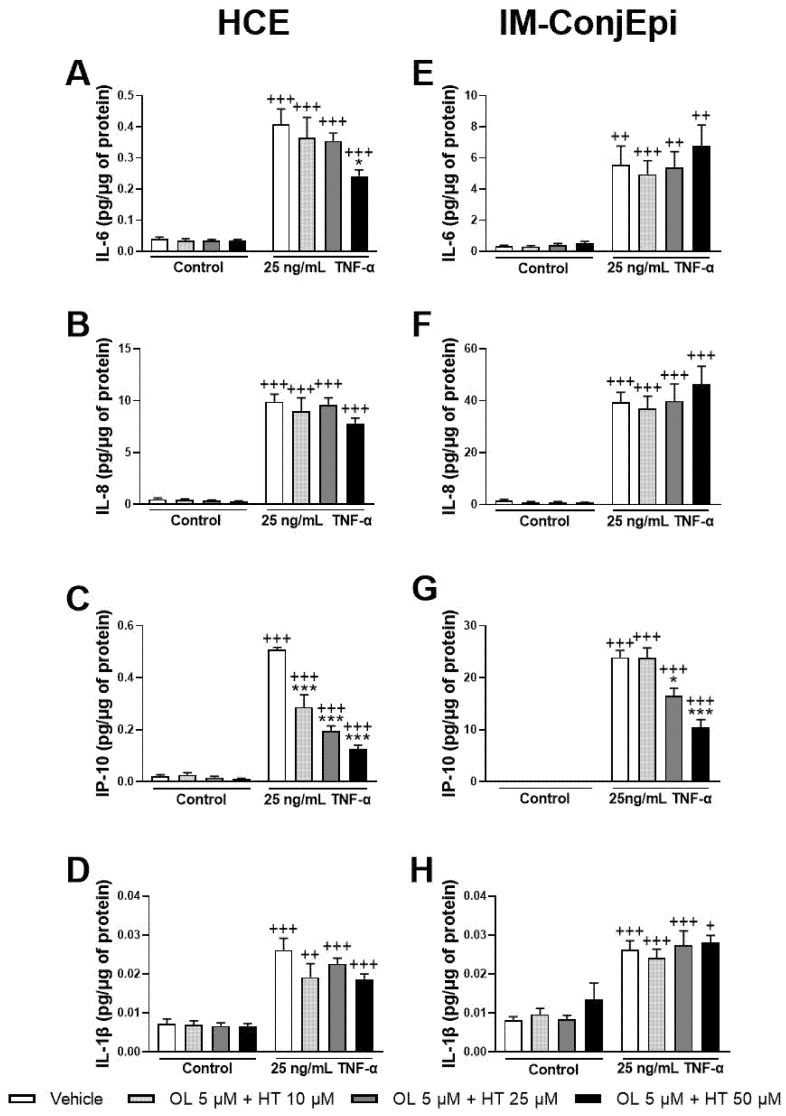
Effect of the mixture of Hydroxytyrosol (HT) and Oleuropein (OL) on TNF-α-induced cytokine release by HCE (**A**–**D**) and IM-ConjEpi (**E**–**H**) cells. Cells were pre-treated with 5 μM of OL + 10 μM of HT, 5 μM of OL + 25 μM of HT, 5 μM of OL + 50 μM of HT, or vehicle (cell culture medium) for 2 h. Following this, they were stimulated with 25 ng/mL TNF-α in the presence of the treatments for 24 h. Vehicle-treated-TNF-α stimulated cells and cells not stimulated with TNF-α were used as control. IL-6, IL-8, IP-10, and IL-1β were measured in cell supernatants by a multiplex bead-based array. For HCE cells, 5 μM of OL + 10 μM of HT had a synergistic effect, decreasing IP-10 levels (**C**), whereas the decrease of IL-6 levels by 5 μM of OL + 50 μM of HT (**A**) can also be achieved by 50 μM HT alone. None of the mixtures tested were able to decrease IL-8 (**B**) or IL-1β (**D**) secretion significantly. For IM-ConjEpi cells, 5 μM of OL + 25 μM of HT reduce IP-10 production (**G**); however, this is also demonstrated by HT alone. None of the mixtures were able to inhibit IL-6 (**E**), IL-8 (**F**), or IL-1β (**H**) secretion significantly. Data are presented as picograms (pg) of cytokine/chemokine per micrograms (μg) of total protein for three independent experiments (performed in duplicate) ± SEM. * *p* < 0.05, *** *p* < 0.001, compared with vehicle-treated-TNF-α stimulated cells; + *p* < 0.05, ++ *p* < 0.01, +++ *p* < 0.001, compared with control cells. Vehicle: Cell culture medium.

The anti-inflammatory effect of different mixtures of low concentrations of the pure phenolic compounds was also studied. The mixture of 5 μM of OL + 10 μM of HT significantly decreased TNF-α-induced IP-10 secretion by HCE cells (Figure 7C), demonstrating a strong synergistic effect. Further, 5 μM of OL combined with 50 μM of HT also inhibited IL-6 production by HCE cells (Figure 7A), and 5 μM of OL with 25 μM of HT decreased IP-10 production by IM-ConjEpi cells (Figure 7G). However, these effects were already demonstrated by HT alone (Figure 4A and Figure 6C).

### 3.4. Antioxidant Activity of Phenolic Solutions

The antioxidant activity of the phenolic solutions was assayed on the UV-B-induced intracellular ROS production on both ocular surface cells, selecting only the non-toxic concentrations as determined by the XTT assay. Same as for the anti-inflammatory activity, a first screening for the effect of the four different OP extracts (at the same concentration, 0.05 mg/mL) was performed on HCE cells. As shown in Figure 2D, all extracts were able to reduce ROS levels by the baseline cells, acting preventively. However, only OPT2 and OPT3 decreased ROS levels significantly in the UV-B-induced cells at the selected concentration, demonstrating a curing antioxidant effect.

**Figure 8:**
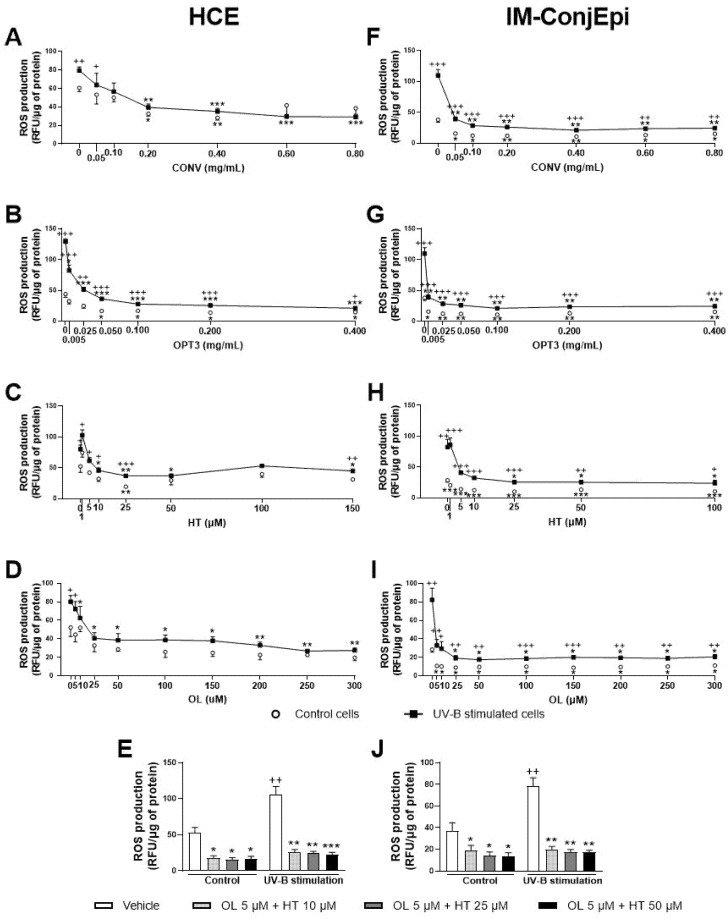
Effect of Hydroxytyrosol (HT), Oleuropein (OL), and their combination, together with the conventional (CONV) and the selected optimized (OPT3) olive pomace (OP) extracts on UV-B-induced reactive oxygen species (ROS) production by HCE HCE (**A**–**E**) and IM-ConjEpi (**F**–**J**) cells. Cells were pre-treated with OL (5, 10, 25, 50, 100, 150, 200, 250, and 300 μM), HT (1, 5, 10, 25, 50, and 100 μM for both cell lines, and 150 μM for HCE), OL + HT (5 μM OL + 10 μM HT, 5 μM OL + 25 μM HT, 5 μM OL + 50 μM HT), CONV (0.05, 0.10, 0.20, 0.40, 0.60, and 0.80 mg/mL), OPT3 (0.005, 0.025, 0.050, 0.100, 0.200, and 0.400 mg/mL), or vehicle (cell culture medium for HT and OL or 0.4% ethanol for the extracts) for 1 h. Subsequently, cells were incubated with 10 μM H2DCF-DA solution for 30 min, and then treated with the treatments and exposed to 107.25 mJ/cm^2^ UV-B light (**A**–**D** and **F**–**I**, black squares; **E**,**J**, black bars). After 1 h of incubation, intracellular fluorescence intensity was measured. Vehicle-treated-UV-B stimulated cells and cells not stimulated with UV-B (**A**–**D** and **F**–**I**, white circles; **E**,**J**, white bars) were used as control. CONV, OPT3, HT, and OL inhibited ROS production by HCE cells at 0.20 mg/mL (**A**), 0.005 mg/mL (**B**), 10 μM (**C**), and 10 μM (**D**), respectively. In this cell line, the combination of 5 μM OL + 10 μM HT demonstrated a synergistic effect in the baseline cells (**E**). For IM-ConjEpi cells, ROS levels were reduced significantly by CONV, OPT3, HT, and OL at 0.05 mg/mL (**F**), 0.005 mg/mL (**G**), 25 μM (**H**), and 25 μM (**I**), respectively. Furthermore, 5 μM of OL + 10 μM of HT had an increased activity on UV-B stimulated IM-ConjEpi cells, compared with each compound alone (**J**). Data are presented as relative fluorescence units (RFU) normalized to micrograms (μg) of total protein for three independent experiments (performed in duplicate) ± SEM. * *p* < 0.05, ** *p* < 0.01, *** *p* < 0.001, compared with vehicle-treated-UV-B stimulated cells; + *p* < 0.05, ++ *p* < 0.01, +++ *p* < 0.001, compared with control cells. Vehicle: Cell culture medium (**E**,**J**).

Further, dose-dependent studies were performed for the antioxidant effect of CONV and OPT3 extracts, together with HT and OL on both HCE and IM-ConjEpi cells. Figure 8 presents the antioxidant activity based on the dose-dependent studies performed for CONV, OPT3, HT, OL, and OL + HT mixtures. In the case of HCE cells, CONV and OPT3 significantly inhibited ROS production, starting from 0.20 mg/mL (Figure 8A) and 0.005 mg/mL (Figure 8B), respectively. It is important to highlight that, for CONV, no significant differences were observed between UV-B exposed and non-exposed cells from 0.10 mg/mL, while OPT3 decreased the ROS levels significantly in non-exposed cells from 0.050 mg/mL. Therefore, both extracts also demonstrate a preventive antioxidant effect. HT was able to inhibit ROS production from 10 μM (except 100 μM), while the concentrations of 50 and 100 μM prevented it (Figure 8C). OL also decreased ROS levels from 10 μM with no significant differences compared with baseline cells, demonstrating a strong curing and preventive antioxidant activity (Figure 8D).

Regarding IM-ConjEpi cells, CONV and OPT3 significantly decreased ROS levels in both UV-B exposed and non-exposed cells, from 0.05 mg/mL (Figure 8F) and 0.005 mg/mL (Figure 8G), respectively. HT and OL also demonstrated a significant antioxidant effect, both from 25 μM, while ROS production was prevented in non-exposed cells from 1 μM for HT (Figure 8H) and 5 μM for OL (Figure 8I).

In terms of OL + HT combination, from low concentrations of 5 μM of OL + 10 μM of HT, the mixture prevented and decreased UV-B-induced intracellular ROS production in both cell lines (Figure 8E,J). The synergistic antioxidant activity can be clearly observed not only in the UV-B exposed IM-ConjEpi cells, but also in the baseline ROS production by HCE cells. The antioxidant effect in IM-ConjEpi non-stimulated cells can be achieved by each compound alone, while the decrease of ROS levels in HCE stimulated cells can be observed by HT alone.

## 4. Discussion

This work proposes the valorization of an environmentally hazardous agro-industrial by-product into an extract with demonstrated potential as treatment for inflammatory and oxidative stress-related ocular surface diseases. Different extracts derived from OP, together with two of their major phenolic compounds, HT and OL, were tested in vitro on human corneal and conjunctival epithelial cells for their cytotoxicity, anti-inflammatory, and antioxidant effect.

Regarding the results of cell viability assay for both cell lines, CONV and OPT1 can be used up to 0.80 mg/mL, while OPT2 and OPT3 up to 0.40 mg/mL. HT is nontoxic up to 100 μM, OL up to 300 μM, and their mixture OL + HT up to 5 μM of OL + 50 μM of HT. These data agree with studies already published regarding cytotoxicity of the same or similar phenolic compounds on ocular cell lines. Granner et al. [30], Zhu et al. [32], and Liu et al. [33] used safely HT up to 100 μM in retinal pigment epithelial cells, while Zou et al. [31] proved the antioxidant effect of HT in the same cell line up to 200 μM. Abengózar-Vela et al. [13] showed that resveratrol is nontoxic up to 300 μM for ocular surface epithelial cells, while for Paladino et al. [42], resveratrol can be safely used up to 200 μM for retinal epithelial cells. Stoddard et al. [43] studied the antioxidant activity of four different polyphenols (quercetin, n-propyl gallate, epigallocatechin gallate (EGCG), and gallic acid) in human corneal cells and demonstrated that their toxic concentrations could vary from 41.3 to 337.5 μM, depending on the compound. Regarding cytotoxicity studies of crude extracts on human corneal cells, *Aloe vera* extract can be used up to 0.13 mg/mL [44], Maple leaves extract up to 0.20 mg/mL [45], and *Polygonum cuspidatum* aqueous extract up to 100 mg/mL [46]. Importantly, as also can be observed from the four different extracts produced from the same material in this study, the extract composition can vary, depending on the extraction method and conditions. This can affect the maximum allowable concentration of each extract.

Our results also showed that, among the four extracts derived from OP tested, CONV (produced by conventional solid–liquid extraction) and OPT3 (generated by sequential scCO_2_ de-oiling, followed by PLE optimization) demonstrated a strong anti-inflammatory activity in a dose-dependent manner. Both inhibited TNF-α -stimulated IL-6, IL-8, IL-1β, and IP-10 secretion by ocular surface corneal and conjunctival epithelial cells. HT was also able to decrease the levels of the aforementioned interleukins in both cell types. OL demonstrated significantly less anti-inflammatory effect than HT or OP extracts, inhibiting only IP-10 stimulated production in conjunctival cells. However, when OL is combined with HT, the mixture can inhibit IP-10 secretion by HCE cells at very low concentrations, at which each compound alone would have no significant activity. This fact proves that the compounds can demonstrate a remarkable synergistic anti-inflammatory effect when combined. Increased levels of the measured cytokines/chemokines have been related to several inflammatory ocular surface diseases, such as DES [47,48,49,50,51,52,53,54,55,56,57] and ocular allergy [58,59,60,61]. More recent findings reveal altered levels of IL-17A in conjunctiva of DES patients, proving that IL-17A plays an important role in the conjunctival epithelial disruption [62]. High levels of IL-17A were also detected in mice stimulated by desiccating stress [49]. However, in our study, TNF-α failed to stimulate IL-17A production in both ocular surface cell lines selected; additionally, in some of our experiments on HCE cells, IL-1β secretion upon TNF-α-stimulation was not sufficient. Another proinflammatory stimulus should be tried in the future either alone or in combination with TNF-α.

In terms of extracts’ composition, as already described in Table 1, CONV contained 1.7 mg of HT/g of DE and 3.4 mg of OL/g of DE, OPT1: 4.4 mg_HT_/g and 5.7 mg_OL_/g, OPT2: 2.9 mg_HT_/g and 11.4 mg_OL_/g, and OPT3: 7.7 mg_HT_/g and 0.0 mg_OL_/g. Hence, at a concentration of 0.40 mg/mL (used for the first screening of anti-inflammatory activity), 4.4 μM of HT and 2.5 μM of OL were tested for CONV, 11.4 μM of HT and 4.2 of μM of OL for OPT1, 7.5 μM of HT and 8.4 μM of OL for OPT2, and 20.0 μM of HT and 0 μM of OL for OPT3. As already mentioned, HT demonstrated a much stronger inhibition of the secretion of the selected interleukins/chemokines than OL on both cell lines. Therefore, because OPT3 had the strongest anti-inflammatory activity, this can be probably related more to HT (or similar compounds) than OL. OPT2 had the highest concentration of OL; however, it demonstrated very poor inhibition of the selected cytokine/chemokines. This fact can also support this hypothesis. On the other hand, OPT3 comprises many different phenolic compounds apart from HT. As can be observed from Table 1, OPT3 is the extract with the highest total phenolic content (2.6 times more than the CONV) and simple phenols’ concentration (like HT) [38]. For OPT3, 0.200 mg/mL comprise 10.0 μM HT and 0 μM OL, while 0.050 mg/mL contains 2.5 μM HT and 0 μM OL. For CONV, 0.20 mg/mL comprises 2.2 μM HT and 1.3 μM OL, 0.40 mg/mL includes 4.4 μM of HT and 2.5 μM of OL, while 0.60 mg/mL contains 6.6 μM of HT and 3.8 μM of OL. Comparing the effective concentrations of OL and HT alone with those included in the concentrations of the extracts tested, a clear synergistic effect can be observed in the extracts. This fact has already been proven by the combination of OL + HT. All these data can explain why OPT3 demonstrate a stronger anti-inflammatory activity compared with CONV, confirming our hypothesis.

In addition, all extracts and compounds tested demonstrated a significant antioxidant activity in a dose-dependent way, starting from very low concentrations, inhibiting UV-B induced ROS production in corneal and conjunctival cells. The combination of OL + HT demonstrated a strong synergistic antioxidant effect in both cell lines either in the stimulated (for IM-ConjEpi) or in the baseline (for HCE) cells. ROS are produced during oxidative metabolism as by-products and are formed by partial reduction of oxygen [63,64]. Oxidative stress is the disruption of the balance between the antioxidant and the pro-oxidant system of the cells, many times leading to oxidative damage [65]. The oxidative damage has been involved in the pathophysiology of many chronic systematic diseases, like cancer, inflammation, and neuro-degenerative disorders [2]. Ocular surface epithelial tissues are exposed to atmospheric oxygen and UV rays of the sunlight [66], also being vulnerable to oxidative stress-induced ocular diseases. Accumulated ROS in tears and Meibomian gland have been related to ocular tissue inflammation and damage [67]. Oxidative damage has been proven to be involved in the pathophysiology of several ocular surface diseases, such as conjunctivochalasis [6], atopic keratoconjunctivitis [7], and Sjogren and non-Sjogren DES [7,9,68,69]. Further, Birkedal-Hansen et al. [70] proved that chronic exposure to environmental stress upregulates oxidative biomarkers, causing loss of the regenerative ability of the corneal cells.

At the concentration used during the first screening of antioxidant activity (0.05 mg/mL), 0.6 μM of HT and 0.3 μM of OL were tested for CONV, 0.18 μM of HT and 0.5 of μM OL for OPT1, 0.9 μM of HT and 1.1 μM of OL for OPT2, and 2.5 μM HT and 0 μM OL for OPT3. OPT3 at 0.005 mg/mL comprises 0.3 μM HT and 0 μM OL. CONV at 0.20 mg/mL contains 2.2 μM HT and 1.3 μM OL, while at 0.05 mg/mL, it contains 0.3 μM of HT and 0.6 μM of OL. HT and OL alone can inhibit ROS production by HCE cells at 10 μM and by IM-ConjEpi at 25 μM. From the combination of OL + HT, a synergistic antioxidant effect was observed. As already explained above, both extracts comprise several phenolic compounds and different total phenolic content (Table 1) [38]. In addition, from the results of both cell lines, it can be observed that OPT3 demonstrates a strong inhibition of the ROS production in 10 to 40 times lower concentrations compared with CONV. Therefore, the synergistic antioxidant activity of several phenolic compounds present in the extracts can be confirmed.

The beneficial anti-inflammatory and antioxidant effects of other pure phenolic compounds on the ocular surface have already been reported. For instance, previous studies from our group demonstrated that quercetin and resveratrol, either alone or in combination, inhibited IL-6, IL-8, and IP-10 secretion by TNF-α–stimulated human corneal and conjunctival cells [13]. They also reduced IL-4, IL-1α, IL-2, and TNF-α tear production in mice exposed to desiccating stress and decreased corneal staining when administrated as eye drops [14]. CD4 T-cell conjunctival infiltration was also decreased in adoptively transferred mice with T cells from desiccating stress-exposed animals treated with quercetin and resveratrol. In addition, both compounds were able to inhibit the UV-B induced oxidative stress in human corneal and conjunctival cells, in a dose-dependent manner [13]. Further, several studies have demonstrated the in vitro anti-inflammatory activity on human corneal cells of curcumin [71], DA-6034 (7-carboxymethyloxy-3′4′,5-trimethoxy flavone) [72], Daidzin [73], EGCG [74,75,76,77], Pterostilbene [78], and a mixture of ferulic acid with kaempferol [79]. Catechin was also able to reduce Prostaglandin E2 production on rabbit cornea cells [80]. A strong antioxidant activity on human corneal cells has been reported only for Pterostiblene [78], Daidzin [73], and EGCG [74]. However, there are few studies exploring and establishing the relation between the antioxidant and the anti-inflammatory activity of natural phenolic compounds on human ocular surface cells. In addition, all these studies examined the effect of the molecules only on corneal cells and not on any additional ocular surface cell line. Our work presents an in vitro comparative study for the olive phenolic compounds between two different parts of the ocular surface (conjunctiva and cornea) exclusively of the human epithelium. Thus, it proposes a promising therapy for oxidative- and inflammatory-related diseases of the entire human ocular surface.

The antioxidant activity of the phenolic compounds has been attributed to the presence of the quinone/semiquinone/hydroquinone triad equilibrium, the chain-breaking scavenging effect of aromatic H atom donors, and the hydroxyl substitution of the aromatic ring [81,82,83]. Regarding the latest, the position and number of the aromatic hydroxyl groups in the structure of the phenolic molecule can affect its final ROS scavenging capacity [84]. The anti-inflammatory activity of these molecules has also been related to the position of these groups. In our study, HT was the molecule with the highest inhibitory activity of the measured ILs. For HT, the hydroxyl groups have been found to demonstrate strong interaction with the ASP348 protein of sirtuin 1 enzyme, upregulating its expression, and hence downregulating inflammatory response by vascular endothelial cells [85]. More recently, hydrogen bond interactions have been confirmed between the hydroxyl groups of HT and the Ser-530 residue of cyclooxygenase-2, inhibiting the expression of the enzyme [86]. It is also important to observe that TY, which does not have the 3-hydroxyl of HT, does not have any affinity with the enzyme, confirming the importance of the position of these groups [86].

Apart from individual phenolic compounds, numerous crude polyphenolic-rich extracts have also demonstrated strong in vitro antioxidant and/or anti-inflammatory activity on the ocular surface. Preliminary studies on fractions of olive polyphenolic extracts have already demonstrated interesting inhibition of ROS and TNF-α production by UV-B-induced and LPS-induced rabbit epithelial corneal cells, respectively [37]. However, our work consists of a complete pharmacological study of four different interleukins/chemokines secretion by two different cell lines of the human epithelium not only for olive extracts, but also for their two major phenolic compounds. Further, it compares extracts with different composition obtained from the same material, explaining their distinct activities. Regarding different plant materials, extracts from *Camellia japonica* [87], *Chamaecyparis obtuse* [88], *Aloe vera* [44], *Euphrasia officinalis* [89], *Lamium album* [90], *Polygonum cuspidatum* [46], and Maple leaves [45] have also been found to reduce inflammatory biomarkers in human corneal cells. Hence, the potential of naturally derived polyphenols and polyphenols-rich extract is becoming an emerging issue in the field.

In summary, our results have demonstrated that a convectional and an HT- enriched optimized extract derived from OP, along with the major OP pure phenolic compounds HT and OL, can inhibit inflammation and oxidative damage in human corneal and conjunctival epithelial cells. However, there are some limitations in this study. Although TNF-α acted good as inflammatory stimulus, different inducers could be used to achieve the stimulation and measurement of IL-17A, TNF-α itself, and/or any other interleukin/chemokine not stimulated by TNF-α. Further, although HCE has been proven to demonstrate high correspondence to the human tissues of origin [39], IM-ConjEpi is a relatively new commercial SV-40 Large T antigen immortalized cell line from primary human conjunctival epithelial. However, according to the manufacturer, it has a 99% purity and maintains expression of epithelial specific markers such as CK18 and CK19. Finally, it is also true that in vivo models are much more complex. For many of the compounds [72,73,76,77,79,91,92,93] and extracts [46,87,88,94,95,96,97] mentioned in the bibliography, the anti-inflammatory activity has also been proven in vivo. For EGCG [98] and Daidzin [73], the in vivo antioxidant effect has also been studied. Hence, in vivo studies in a desiccating stress mice model are currently being performed for selected extracts and compounds, to support our in vitro findings.

In conclusion, extracts derived from OP and their major phenolic compounds, particularly HT, seem to be promising potential treatment for ocular surface inflammatory diseases, such as DES. The use of these type of OP extracts is of chief importance for the green development of related industries, as they propose a high value application of an agro-industrial by-product. The results of this study illustrate how the sustainable and intensified extraction techniques are proved to be competitive compared to the conventional ones, while a high selectivity towards biomarkers related to dry eye is established.

## 5. Patents

The results are patent pending.

## Figures and Tables

**Table 1:** Characterization of the conventional (CONV) and the three optimized (OPT1, OPT2, and OPT3) olive pomace (OP) extracts in terms of extraction yield (EY) (as mg of dry extract (DE)/g of DRY OP), oxygen radical absorbance capacity antioxidant activity (ORAC-AA) (as mmol of Trolox equivalents (TE)/g of DE), and total phenolic content (TPC) (as mg of gallic acid equivalents (GAE)/g of DE), as well as extract richness in Hydroxytyrosol (HT), Oleuropein (OL) and Tyrosol (TY) (as mg of compound/g of DE). The results are presented as average ± standard deviation.

Extract	Method	EY (mg_DE_/g_DRY OP_)	ORAC-AA (mmol_TE_/g_DE_)	TPC (mg_GAE_/g_DE_)	HT (mg_HT_/g_DE_)	OL (mg_OL_/g_DE_)	TY (mg_TY_/g_DE_)
CONV	Conventional solid/liquid	94 ± 6	4.36 ± 0.08	131 ± 27	1.7 ± 0.7	3.4 ± 0.5	1.9 ± 0.3
OPT1	Pressurized liquid extraction	8.0 ± 1.6	8.8 ± 0.9	282 ± 6	4.4 ± 0.7	5.7 ± 0.8	4.1 ± 0.8
OPT2		91 ± 10	4.09 ± 0.01	259.11 ± 0.01	2.9 ± 0.3	11.4 ± 1.2	1.3 ± 0.2
OPT3		56 ± 7	5.1 ± 0.2	336 ± 18	7.7 ± 0.7	0.0 ± 0.0	4.1 ± 0.2

## Abbreviations

ANOVA	Analysis of Variances
BCA	Bicinchoninic Acid
CONV	Conventional extract
DE	Dry Extract
DES	Dry Eye Syndrome
DMEM/F-12	Dulbecco’s Modified Eagle’s Medium/Nutrient Mixture F-12
DMSO	Dimethyl Sulfoxide
DPBS	Dulbecco’s Phosphate Buffered Saline
EGCG	Epigallocatechin Gallate
EGF	Epithelial Growth Factor
EtOH	Ethanol
FBS	Fetal Bovine Serum
FD-OP	Freeze-Dried Olive Pomace
GAE	Gallic Acid Equivalents
H2DCF-DA	20,70-dichlorodihydrofluorescein diacetate
HCE	Human Corneal Epithelial cells
HT	Hydroxytyrosol
IL	Interleukin
IM-ConjEpi	Immortalized human Conjunctival Epithelial cells
IP	Interferon γ-induced protein
LFU	Lacrimal Functional Unit
OL	Oleuropein
OL+HT	Combination of Oleuropein and Hydroxytyrosol
OP	Olive Pomace
OPT	Optimized extracts
ORAC	Oxygen Radical Absorbance Capacity
ORAC-AA	Oxygen Radical Absorbance Capacity Antioxidant Activity
PLE	Pressurized Liquid Extraction
PMS	5-Methylphenazinium Methyl Sulfate
ROS	Reactive Oxygen Species
scCO_2_	Supercritical Carbon CO_2_
SEM	Standard Error of the Mean
TE	Trolox Equivalents
TNF	Tumour Necrosis Factor
TPC	Total Phenolic Content
TY	Tyrosol
UV	Ultraviolet
XTT	2,3-Bis-(2-Methoxy-4-Nitro-5-Sulfophenyl)-2H-Tetrazolium-5-Carboxanilide
